# Mechanisms of mitochondrial dysfunction and protective strategies in skin flap ischemia-reperfusion injury

**DOI:** 10.3389/fphar.2026.1865091

**Published:** 2026-06-25

**Authors:** Quan Shi, Zairong Wei

**Affiliations:** 1 Department of Burns and Plastic Surgery, Affiliated Hospital of Zunyi Medical University, Zunyi, China; 2 The Collaborative Innovation Center of Tissue Damage Repair and Regeneration Medicine, Zunyi Medical University, Zunyi, China; 3 Guizhou Biofabrication Laboratory, Affiliated Hospital of Zunyi Medical University, Zunyi, China; 4 Department of Breast, Thyroid and Vascular Surgery, The Affiliated Traditional Chinese Medicine Hospital, Southwest Medical University, Luzhou, China

**Keywords:** flap ischemia-reperfusion injury, mitochondrial homeostasis, mitochondrial quality control, oxidative stress, programmed cell death, tissue repair

## Abstract

Flap transplantation remains a cornerstone of reconstruction of complex tissue defects and restoration of local form and function. However, ischemia-reperfusion (I/R) injury continues to compromise flap viability and is a major cause of distal necrosis. Emerging evidence suggests that mitochondria are among the earliest and most severely affected organelles during flap I/R, placing them at the center of tissue injury. Once disrupted, mitochondrial dysfunction may aggravate microcirculatory failure, amplify inflammatory responses, and accelerate tissue damage through excessive mitochondrial reactive oxygen species (mtROS) generation, mitochondrial permeability transition pore (mPTP) opening, loss of mitochondrial membrane potential, and impaired mitochondrial quality control. Disruption of mitochondrial homeostasis also reshapes the behavior of endothelial cells, macrophages, fibroblasts, and vascular smooth muscle cells, thereby influencing flap repair outcomes. In this review, we focus on mitochondrial homeostasis as a unifying framework for understanding flap I/R injury. We discuss its involvement in oxidative stress, calcium overload, mPTP opening, metabolic dysfunction, defective mitochondrial quality control, and mitochondria-related programmed cell death. We further summarize recent therapeutic strategies designed to preserve or restore mitochondrial homeostasis, with the goal of informing future approaches to improve flap survival and tissue repair.

## Graphical Abstract

**Figure:**
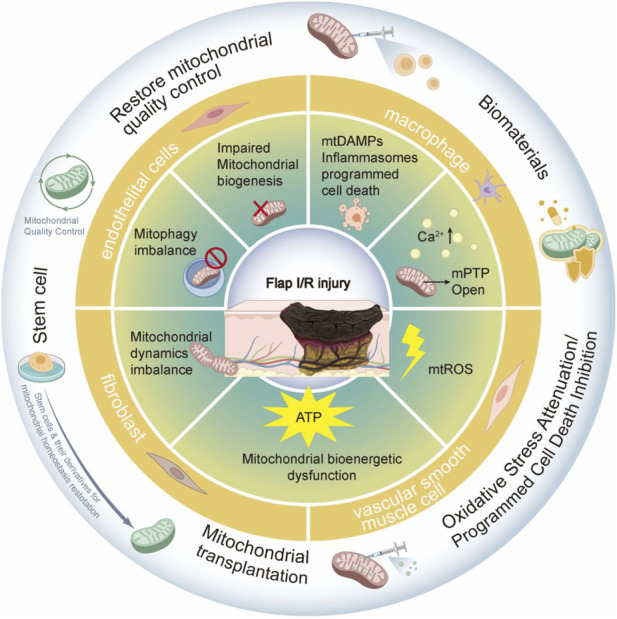

## 1 Introduction

Flap transplantation is a fundamental reconstructive approach for repairing complex soft-tissue defects and restoring both form and function. Despite continuous technical advances, flap necrosis and partial flap loss remain important clinical problems. Reported necrosis rates vary across flap types, ranging from 7.78% to 9.15% in free flaps, 2.1%–13.6% in propeller perforator flaps, and 7%–40% in breast reconstruction-related flaps (Serra et al., 2024; Matarazzo et al., 2025; Bovill et al., 2023; Weidman and Parikh, 2026). These data suggest that flap survival remains clinically challenging despite advances in surgical techniques and perioperative management. Increasing evidence suggests that ischemia-reperfusion (I/R) injury is a major determinant of flap failure across multiple reconstructive settings, including random-pattern flaps, free flaps, and perforator flaps (Yeou and Shin, 2026; Drysch et al., 2025; Jia et al., 2025). Although reperfusion is necessary to restore tissue perfusion, it also initiates oxidative stress, inflammatory amplification, cell death, and defective tissue repair, all of which further compromise distal flap viability (Yeou and Shin, 2026; Drysch et al., 2025). Effective and reliable clinical strategies to limit this process, however, remain lacking.

In recent years, research on flap I/R injury has moved beyond a simple focus on inadequate blood flow recovery toward a broader view that includes organelle-centered mechanisms of injury (Liu Z. et al., 2025). Among the organelles involved, mitochondria appear to be one of the earliest and most severely affected. Mitochondrial dysfunction may drive mtROS accumulation, loss of mitochondrial membrane potential, and opening of the mitochondrial permeability transition pore, thereby worsening microcirculatory dysfunction, inflammation, and programmed cell death and ultimately aggravating tissue necrosis (Jia et al., 2025; Liu S. et al., 2025). Accordingly, strategies aimed at restoring mitochondrial homeostasis have become an important direction in efforts to improve flap survival and promote tissue repair.

To date, increasing evidence has supported the protective value of mitochondria-targeted interventions from several angles, including suppression of oxidative stress, improvement of energy metabolism, regulation of mitochondrial quality control, and inhibition of programmed cell death (Chen et al., 2022; Lee et al., 2024; Jeon et al., 2025; Yang et al., 2025a; Pan et al., 2025). In this context, the present review focuses on disruption of mitochondrial homeostasis in flap ischemia-reperfusion injury. It discusses the roles of mitochondria in oxidative stress, calcium overload and mPTP opening, energy metabolic dysfunction, mitochondrial quality control imbalance, and programmed cell death. It also examines the pathological alterations in endothelial cells, macrophages, fibroblasts, and vascular smooth muscle cells associated with mitochondrial injury (Grossini et al., 2025; Liu G. et al., 2025; Bansal et al., 2024; Qin et al., 2023). On this basis, the review further summarizes representative intervention strategies targeting mitochondrial homeostasis that have emerged in recent years, with the aim of providing a useful reference for mechanistic studies of flap protection and development of new therapeutic approaches.

## 2 Key pathological mechanisms of mitochondrial dysfunction-mediated flap I/R injury

**FIGURE 1:**
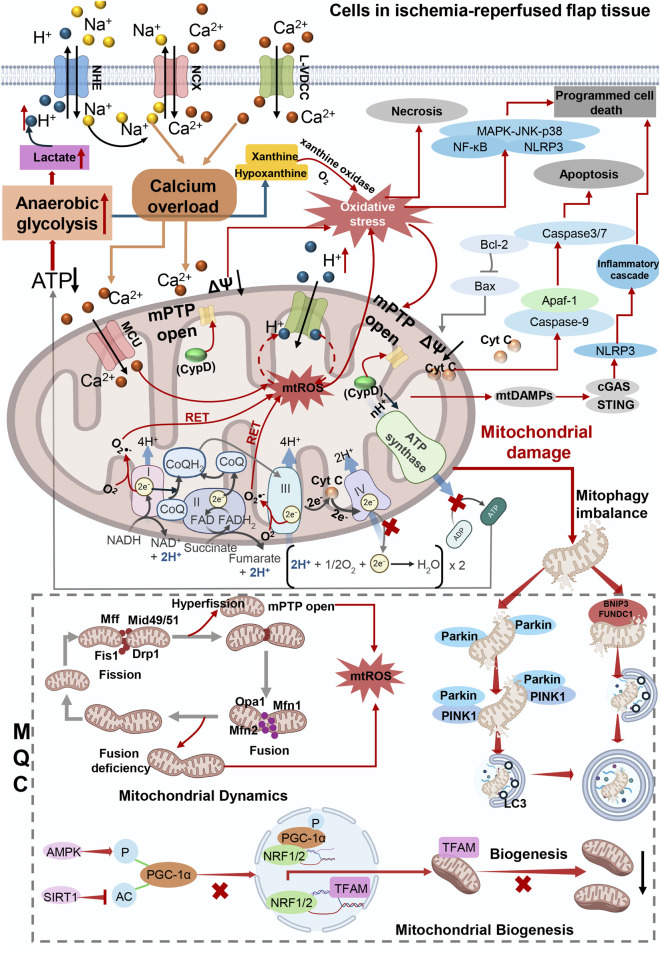
Schematic illustration of the key pathological mechanisms of mitochondrial dysfunction-mediated flap ischemia-reperfusion injury.

Mitochondrial dysfunction-mediated key pathological mechanisms contribute to flap I/R injury, as illustrated in Figure 1.

### 2.1 Mitochondrial bioenergetic impairment

When the flap undergoes ischemia and hypoxia, mitochondrial oxidative phosphorylation is one of the earliest processes to be impaired, because oxygen, the terminal electron acceptor of the electron transport chain, becomes abruptly limited. As a result, the upstream respiratory complexes and the coenzyme Q pool become progressively over-reduced, reoxidation of NADH and FADH_2_ is hindered, the transmembrane proton gradient rapidly dissipates, and ATP production declines markedly. Under these conditions, cells are forced to rely on inefficient anaerobic glycolysis, leading to lactate and H^+^ accumulation and thereby exacerbating acidosis and metabolic imbalance (Alotaibi et al., 2025; Pham et al., 2024; Xu X. et al., 2025).

With reperfusion, oxygen and metabolic substrates return to the tissue, but mitochondrial function does not immediately normalize. Instead, the highly reduced state established during ischemia, together with the buildup of metabolic intermediates, leaves the electron transport chain in a maladaptive state during early reoxygenation. In particular, accumulated succinate is rapidly oxidized, driving a large influx of electrons through complex II into the coenzyme Q pool. When membrane potential is rapidly re-established while the coenzyme Q pool remains highly reduced, the highly reduced coenzyme Q pool and restored proton motive force can drive reverse electron transport through complex I, rather than normal forward electron transfer, thereby increasing pathological electron leakage and superoxide generation at complex I (Zhang C. et al., 2025; Rozich et al., 2025; Fang et al., 2025). However, direct evidence demonstrating reverse electron transport in flap ischemia-reperfusion injury remains limited. Therefore, this mechanism should be regarded as a plausible explanation mainly inferred from studies in other ischemia-reperfusion models, particularly cardiac and cerebral ischemia-reperfusion injury. Given the shared metabolic features among ischemic tissues, including succinate accumulation, mitochondrial over-reduction, and abrupt reoxygenation, reverse electron transport may also contribute to early mitochondrial ROS production in flap ischemia-reperfusion injury. Thus, ischemia is characterized primarily by excessive reduction of the electron transport chain and ATP depletion, whereas reperfusion further amplifies early metabolic injury through maladaptive restoration of oxidative phosphorylation and reverse electron transport-driven mitochondrial ROS generation, ultimately promoting the progression of flap ischemia-reperfusion injury.

### 2.2 mtROS overproduction

Against the background of a highly reduced respiratory chain during flap ischemia, reintroduction of oxygen in the early reperfusion phase does not immediately restore mitochondrial function. Instead, it triggers a rapid burst of mitochondrial reactive oxygen species (mtROS), which represents a major amplification event in ischemia-reperfusion injury (Alotaibi et al., 2025). Under I/R conditions, succinate that accumulates during ischemia is rapidly oxidized upon reperfusion, driving a large influx of electrons through complex II into the coenzyme Q pool. When the coenzyme Q pool remains highly reduced while mitochondrial membrane potential is rapidly re-established, the restored proton motive force can drive reverse electron transport through complex I, thereby promoting excessive superoxide generation at complex I. At the same time, electron leakage from the Qo site of complex III provides another important source of mtROS (Xu X. et al., 2025; Rozich et al., 2025; Bao et al., 2025).

Oxidative stress in early reperfusion is not derived from mitochondria alone. Reactive oxygen species (ROS) generated by xanthine oxidase and NADPH oxidase can further damage mitochondrial membranes and respiratory chain complexes, thereby increasing electron leakage. In turn, these non-mitochondrial ROS signals can act together with mtROS to form a feed-forward cycle that amplifies ROS production from multiple sources (Jia et al., 2025; Cipriano et al., 2023). When mtROS rise excessively, the inner mitochondrial membrane becomes a major target of injury. Oxidation of cardiolipin can directly destabilize respiratory chain complexes, disrupt inner membrane integrity, and reduce electron transfer efficiency. Meanwhile, mtROS also acts as a signaling mediator that sustains activation of NF-κB, MAPK/JNK/p38, and NLRP3 inflammasome-related pathways, thereby promoting inflammatory mediator release and programmed cell death (Chai et al., 2025; Yang et al., 2025b; Paik et al., 2025; Yang L. et al., 2025; Rius-Pérez et al., 2023).

### 2.3 Ca^2+^ overload and mPTP opening

When mitochondrial reactive oxygen species remain elevated, intracellular Ca^2+^ homeostasis becomes increasingly difficult to maintain, which further intensifies oxidative stress and causes direct mitochondrial injury. During ischemia, ATP depletion suppresses Na^+^/K^+^-ATPase and Ca^2+^-ATPase activity, leading to membrane depolarization, Na^+^ accumulation, and intracellular acidosis. This favors increased Na^+^/H^+^ exchange and secondary dysregulation of Na^+^/Ca^2+^ exchange, with a consequent rise in cytosolic Ca^2+^. After reperfusion, Ca^2+^ entry through the plasma membrane, Ca^2+^ release from the endoplasmic or sarcoplasmic reticulum, and mitochondrial Ca^2+^ uptake all increase, driving mitochondria away from physiological Ca^2+^ buffering and toward pathological Ca^2+^ overload (Yang et al., 2025b; Pagliaro et al., 2026; Guo et al., 2024; Liu et al., 2024).

Once mitochondrial Ca^2+^ entry exceeds the capacity of MCU-dependent uptake and NCLX-mediated efflux to remain balanced, matrix Ca^2+^ accumulation continues to increase. At the same time, removal of low-pH inhibition during reperfusion makes it more likely that Ca^2+^ overload and oxidative damage will synergistically induce mPTP opening (Murphy and Eisner, 2024; Liu Y. et al., 2025). The mPTP is a high-conductance, nonselective channel located in the inner mitochondrial membrane and is highly responsive to Ca^2+^ burden, ROS, inorganic phosphate, and membrane potential changes. During reperfusion, mitochondrial Ca^2+^ accumulation, ROS elevation, and ATP depletion together reduce the threshold for pore opening, with cyclophilin D acting as a central regulator (Morciano and Pinton, 2025). Sustained mPTP opening then results in dissipation of mitochondrial membrane potential, interruption of oxidative phosphorylation, matrix swelling, and eventual rupture of the outer membrane. These events lead to release of pro-apoptotic molecules, including cytochrome c and AIF, markedly impair post-reperfusion cellular recovery, and promote both apoptosis and necrosis-like death, thereby potentially aggravating distal flap necrosis. Accordingly, mPTP opening is now considered a key therapeutic target in studies of mitochondrial protection (Morciano and Pinton, 2025; Singh, 2025), although its direct therapeutic validation in flap I/R remains insufficient.

### 2.4 Mitochondrial dynamics imbalance

Mitochondrial dynamics is a central component of mitochondrial quality control and depends on the balance between fission and fusion. Under physiological conditions, Drp1 mediates mitochondrial fission after being recruited by outer membrane receptors such as MFF, FIS1, and MiD49/51. By contrast, Mfn1/2 and OPA1 regulate fusion of the outer and inner mitochondrial membranes, respectively. Together, these proteins maintain mitochondrial network continuity, cristae integrity, and renewal of the functional mitochondrial pool (Zhang T. et al., 2025; He et al., 2025).

Under ischemia-reperfusion stress, this dynamic balance is disrupted. Excessive Drp1 activation and mitochondrial translocation enhance fission, whereas downregulation of Mfn1/2 and dysregulated OPA1 cleavage suppress fusion. These changes not only promote mitochondrial fragmentation, but also further compromise membrane potential maintenance, respiratory chain coupling, and ATP production (Zhang C. et al., 2025; Fang et al., 2025).

In flap ischemia-reperfusion (I/R) injury, increased Drp1 expression is closely associated with mitochondrial dysfunction and poor flap survival, whereas inhibition of aberrant Drp1 activation can improve flap outcome. In addition, Rg1 has been shown to improve flap perfusion and reduce necrosis by upregulating Mfn2 and downregulating Drp1, further supporting the view that restoration of the fission-fusion balance is important for re-establishing mitochondrial homeostasis and improving flap survival (He et al., 2025; Wang et al., 2021). Overall, mitochondrial dynamics imbalance in flap I/R is characterized mainly by excessive fission and insufficient fusion, leading to accumulation of damaged mitochondria, loss of physiological function, and progressive transition of flap tissue from metabolic vulnerability to irreversible injury.

### 2.5 Mitophagy imbalance

Mitophagy is a core component of mitochondrial quality control and primarily serves to selectively remove depolarized or damaged mitochondria, thereby preserving the integrity of the functional mitochondrial pool (Zhang T. et al., 2025; Uoselis et al., 2023; Wang et al., 2023). When mitochondrial membrane potential declines, PINK1 accumulates on the outer mitochondrial membrane and recruits Parkin, which promotes ubiquitination of outer membrane proteins. Damaged mitochondria are then connected to LC3-positive autophagic membranes through adaptor proteins such as p62, OPTN, and NDP52, allowing their delivery to lysosomes for degradation. In addition to the PINK1/Parkin pathway, receptors including BNIP3, NIX, and FUNDC1 can also directly mediate mitophagy (Yao B.-F. et al., 2024; Liu M. et al., 2025; Yang et al., 2024; Bruqi and Strappazzon, 2025; Shan et al., 2025).

During ischemia-reperfusion, mitophagy shows a biphasic response. Moderate activation helps remove damaged mitochondria in a timely manner and improves tissue tolerance, whereas impaired autophagic flux leads to persistent retention of dysfunctional mitochondria and aggravates injury. By contrast, if mitophagy remains chronically or excessively activated without parallel recovery of mitochondrial biogenesis, the functional mitochondrial pool is further depleted and the energy crisis worsens (He et al., 2025; Wang et al., 2023; Civiletto et al., 2025). Mechanistically, this protective-to-detrimental transition may depend on the severity and duration of mitochondrial damage, the integrity of autophagic-lysosomal flux, and the balance between mitochondrial clearance and biogenesis. Transient mitochondrial depolarization may support selective PINK1/Parkin-dependent removal of damaged mitochondria, whereas sustained mitochondrial injury can promote persistent mitophagy and excessive mitochondrial clearance (Shen et al., 2021; Xu et al., 2024). Moreover, when lysosomal degradation is impaired or PGC-1α/NRF1/TFAM-mediated mitochondrial biogenesis is insufficient, enhanced mitophagy may fail to restore mitochondrial homeostasis and instead accelerate mitochondrial depletion and ATP insufficiency (Li et al., 2025; Liu et al., 2023).

In flap research, increasing evidence indicates that improved flap survival depends not simply on enhancing or inhibiting mitophagy, but on restoring balanced and complete mitophagic flux. Enhancement of Parkin- or TFEB-mediated mitophagy has been shown to reduce oxidative injury and apoptosis, thereby improving flap survival. Conversely, under severe ischemic stress, suppression of excessive PINK1/Parkin-dependent mitophagy may also be beneficial for flap survival (Chen et al., 2022; Pan et al., 2025; Wang et al., 2021; Xue et al., 2023; Zhou et al., 2023). These findings suggest that the role of mitophagy in flap I/R is context-dependent: early or moderate activation may remove ROS-generating mitochondria, whereas prolonged activation combined with defective lysosomal clearance or insufficient mitochondrial renewal may aggravate bioenergetic collapse. Thus, mitophagy imbalance in flap ischemia/reperfusion (I/R) should not be viewed only as insufficient or excessive autophagy, but as a broader disruption involving recognition, sequestration, degradation, and compensatory renewal of damaged mitochondria.

### 2.6 Impaired mitochondrial biogenesis

Within the mitochondrial quality control system, mitochondrial biogenesis serves as the replenishment step that follows removal of damaged mitochondria. This process is mainly governed by the PGC-1α-NRF1/2-TFAM axis. Through activation of NRF1/2 and upregulation of TFAM, PGC-1α promotes mitochondrial DNA (mtDNA) replication, transcription, and renewal of respiratory chain-related proteins, thereby supporting the generation of newly functional mitochondria (Cao et al., 2025; Hwang et al., 2022; Yang et al., 2025d). Mitochondrial biogenesis therefore affects not only mitochondrial abundance, but also oxidative phosphorylation capacity and restoration of energy metabolic homeostasis after tissue injury.

In flap ischemia-reperfusion injury, sustained metabolic stress, oxidative damage, and inflammation suppress the expression and activity of PGC-1α, NRF1/2, and TFAM. This occurs in part through attenuation of AMPK-SIRT1-PGC-1α signaling, leaving damaged mitochondria cleared but newly functional mitochondria insufficiently replenished (Zhang C. et al., 2025; Hao et al., 2025). This defect is especially relevant in distal flap tissue, where perfusion is poor, metabolic stress is high, and reparative demand is increased. Under these conditions, restoration of blood flow alone is often not enough to reverse the ongoing energy deficit and defective repair.

Jeon et al. showed that a sustained oxygen-releasing hydrogel improved local oxygen supply while enhancing PGC-1α-related mitochondrial biogenesis. This was accompanied by increased mitochondrial abundance, greater antioxidant capacity, improved angiogenesis, and better distal flap survival. These findings support restoration of mitochondrial biogenesis as an important strategy for improving flap survival (Yeou and Shin, 2026; Jeon et al., 2025; D’Egidio et al., 2025).

### 2.7 Release of mtDAMPs and inflammatory amplification

In flap ischemia-reperfusion injury, the consequences of mitochondrial damage are not limited to impaired bioenergetics. More importantly, mitochondrial injury can drive amplification of sterile inflammation. Liu et al. noted that the progression of flap ischemia-reperfusion (I/R) is not caused by a single injurious event, but by the interaction between inflammatory responses and multiple forms of programmed cell death, which together promote expansion of distal tissue necrosis (Liu S. et al., 2025). After mitochondrial damage, mitochondrial-derived danger-associated molecular patterns (mtDAMPs), including oxidized mtDNA, cardiolipin, N-formyl peptides, and ATP, can be released and sensed by the innate immune system. Brooks et al. highlighted that mtDAMPs provide an important molecular link between mitochondrial injury and inflammatory activation, and that their release can convert localized cellular damage into a broader innate immune response (Brooks et al., 2026).

Chen et al. further suggested that mtDAMPs can activate signaling pathways such as cGAS-STING and the NLRP3 inflammasome, thereby promoting maturation and release of inflammatory mediators and sustaining local inflammation (Chen K.-Q. et al., 2025). Thus, in flap I/R, mtDAMPs may be not only markers of mitochondrial injury, but also active mediators that couple mitochondrial dysfunction to inflammatory amplification and tissue deterioration, although direct flap-specific validation remains limited.

### 2.8 Mitochondria-related programmed cell death

Mitochondrial damage and inflammatory amplification are major drivers of flap ischemia-reperfusion (I/R) injury and promote disease progression through multiple forms of programmed cell death. Liu et al. noted that pyroptosis, apoptosis, and ferroptosis act as key executional events in flap I/R injury and together provide an important pathological link between inflammatory amplification and expansion of tissue necrosis (Liu S. et al., 2025). Among these processes, inflammasome-related pyroptosis has become a major focus of recent flap research. Studies have shown that both thymoquinone and Shuxuetong injection can reduce pyroptotic injury and improve flap survival by inhibiting NF-κB/NLRP3-related signaling (Yang et al., 2025a; Wang K. et al., 2025). Ferroptosis also contributes to flap necrosis. Exendin-4 has been reported to suppress ferroptosis through upregulation of GPX4, whereas osthole improves flap survival not only by inhibiting ferroptosis through the Nrf2/SLC7A11/GPX4 axis, but also by reducing pyroptosis through inhibition of the NLRP3/caspase-1/GSDMD pathway (Yu et al., 2024; Xu P. et al., 2025).

### 2.9 Mitochondria-endoplasmic reticulum contact site dysfunction

Mitochondria-endoplasmic reticulum contact sites, also termed mitochondria-associated endoplasmic reticulum membranes or mitochondria-associated membranes, are specialized inter-organelle interfaces that coordinate Ca^2+^ transfer, lipid exchange, mitochondrial dynamics, mitophagy, endoplasmic reticulum stress, and cell death signaling (Jiang et al., 2023). In ischemia-reperfusion injury, abnormal MAM remodeling may amplify mitochondrial Ca^2+^ overload, ROS production, mPTP opening, membrane potential collapse, and apoptosis, especially through MAM-enriched Ca^2+^ transfer complexes such as IP3R-GRP75-VDAC (Chen C. et al., 2025; Bertero et al., 2024). Recent studies in renal and cardiac ischemia-reperfusion models further suggest that MAM-associated proteins such as MFN2 and DIAPH1 can regulate ER-mitochondria coupling and influence mitochondrial injury, indicating that MAM homeostasis, rather than simple enhancement or disruption of ER-mitochondria contacts, is critical for mitochondrial protection (Li et al., 2024; Kirshenbaum et al., 2024).

Although direct evidence in flap ischemia-reperfusion injury remains limited, MAM dysfunction may be highly relevant to flap tissue damage. During flap reperfusion, endothelial cells, fibroblasts, vascular smooth muscle cells, and inflammatory cells are exposed to abrupt reoxygenation, Ca^2+^ overload, oxidative stress, and ER stress. Dysregulated ER-mitochondria communication may therefore contribute to endothelial apoptosis, microvascular dysfunction, inflammatory amplification, impaired mitochondrial quality control, and progressive distal necrosis. Future flap-specific studies should directly evaluate MAM ultrastructure, key tethering proteins such as IP3R, GRP75, VDAC1, MFN2, and FUNDC1, mitochondrial Ca^2+^ flux, ROS generation, mPTP opening, perfusion recovery, and necrotic area.

## 3 Effects of mitochondrial dysfunction on key effector cells in flap I/R

**FIGURE 2:**
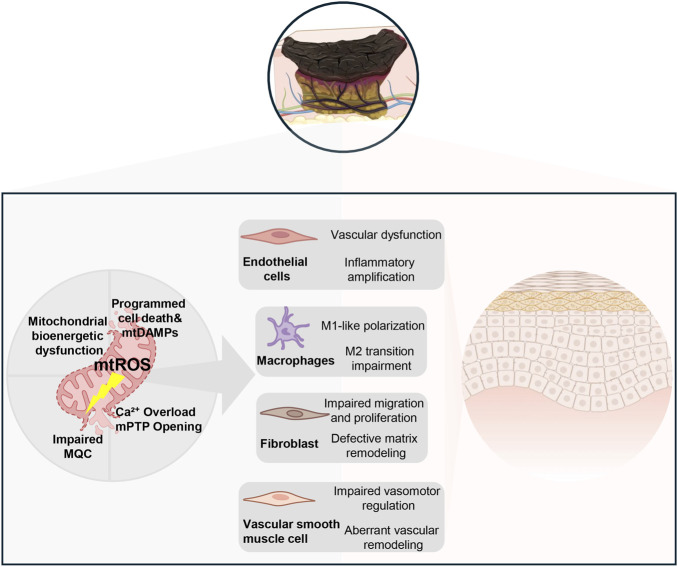
Mitochondrial dysfunction in endothelial cells, macrophages, fibroblasts, and vascular smooth muscle cells contributes to flap necrosis.

These mitochondrial dysfunction–driven alterations in endothelial cell function are summarized in Figure 2.

### 3.1 Endothelial cells

Endothelial cells are key effectors in maintaining flap microcirculatory homeostasis, and their functional state directly influences reperfusion quality, vascular permeability, and inflammatory cell recruitment. Although endothelial cells rely mainly on glycolysis for energy production, mitochondria remain essential for redox regulation, Ca^2+^ signaling, vascular homeostasis, and control of the angiogenic phenotype (Grossini et al., 2025; Luo et al., 2024; Cannito et al., 2025).

Under ischemia-reperfusion stress, mitochondrial dysfunction markedly weakens the ability of endothelial cells to regulate the microcirculation, leading to reduced vascular reactivity, barrier disruption, and impaired reparative capacity. Studies have shown that mitochondrial abnormalities are closely associated with defective endothelial angiogenesis, migration, and tube formation. They also reduce nitric oxide bioavailability and vasodilatory function while promoting a pro-inflammatory adhesive phenotype and increased vascular permeability. These changes facilitate leukocyte recruitment and transendothelial migration, thereby aggravating local inflammation and microcirculatory disturbance (Luo et al., 2023; Wang and He, 2024; Kopych et al., 2025; Klein, 2025). In the distal marginal perfusion zone of the flap, endothelial mitochondrial dysfunction therefore not only compromises blood flow regulation and angiogenic capacity, but may also drive the transition from reversible ischemia to persistent perfusion failure through barrier breakdown and inflammatory amplification.

### 3.2 Macrophages

Macrophages are key immune effectors that regulate inflammatory responses and support tissue repair after flap ischemia-reperfusion injury. Their phenotypic transition and functional activity are closely dependent on mitochondrial metabolic status. When mitochondrial homeostasis is disturbed, macrophages typically show reduced oxidative phosphorylation, accumulation of mitochondrial reactive oxygen species (mtROS), and abnormal immunometabolic reprogramming. These changes favor a pro-inflammatory M1-like phenotype while limiting the transition toward a pro-reparative M2-like phenotype (Cai et al., 2023; Ao-Di et al., 2024; Kumar et al., 2024).

Studies have shown that mitochondrial dysfunction in macrophages not only increases release of pro-inflammatory mediators and amplifies inflammatory signaling, but also impairs phagocytic clearance, resolution of inflammation, and re-establishment of a reparative microenvironment. As a result, tissue repair after injury is compromised (Kumar et al., 2024; Zhao et al., 2025). In flap ischemia-reperfusion injury, macrophage mitochondrial dysfunction therefore intensifies local inflammation and interferes with the transition from inflammation to repair, ultimately affecting flap survival and tissue regeneration.

### 3.3 Fibroblasts

Fibroblasts are important effector cells in maintaining dermal structural integrity during flap repair. Their main functions include migration, proliferation, and synthesis and remodeling of extracellular matrix components such as collagen. These activities support granulation tissue formation, wound contraction, and overall tissue stability. Mitochondrial homeostasis is essential for fibroblasts to maintain energy metabolism, redox balance, and adaptive stress responses. When mitochondrial function is impaired, the associated metabolic and signaling networks are disturbed, leading to persistent defects in coordinated wound repair (Zhao et al., 2025; Xiong et al., 2025; Fang and Lan, 2023).

Loss of mitochondrial homeostasis typically reduces fibroblast migratory, proliferative, and matrix-remodeling capacity. In particular, abnormalities in mitochondrial dynamics can directly impair migration toward the wound bed. Mitochondrial dysfunction can also suppress collagen secretion and extracellular matrix remodeling (Bansal et al., 2024; Zaccaron et al., 2024). As a result, mitochondrial dysfunction in fibroblasts not only delays wound repair, but also compromises the regenerative quality of flap tissue by weakening collagen production and matrix remodeling.

### 3.4 Vascular smooth muscle cells

Vascular smooth muscle cells (VSMCs) are key effectors in maintaining vascular wall stability and regulating vascular tone, and their functional state directly affects local perfusion control. Mitochondrial homeostasis is essential in VSMCs not only for energy supply, but also for maintenance of phenotype, metabolic regulation, and adaptation to stress. When mitochondrial metabolism becomes dysregulated, VSMCs may shift from a contractile phenotype to a synthetic phenotype, thereby increasing proliferative, migratory, and matrix-remodeling activity (Qin et al., 2023; Pearce, 2024).

Mitochondrial dysfunction in VSMCs typically leads to reduced contractility and impaired vascular reactivity, accompanied by downregulation of contractile proteins and phenotypic switching. In addition, disruption of Ca^2+^ microdomain signaling and mitochondrial Ca^2+^ handling can further weaken vasomotor control and promote abnormal vascular remodeling (Yap et al., 2024; Suzuki, 2025; Lu et al., 2024). After flap ischemia-reperfusion, these changes may impair regulation of vascular tone, reduce the efficiency of blood flow redistribution, and drive maladaptive vascular remodeling. Together, they hinder perfusion recovery in the ischemic marginal zone and compromise flap survival.

## 4 Protective strategies for restoring mitochondrial homeostasis in flap I/R

In flap ischemia-reperfusion injury, recent work has moved beyond a narrow emphasis on microcirculatory disturbance to address deeper mechanisms, including oxidative stress, inflammation, cell death, and mitochondrial dysfunction (Drysch et al., 2025). In clinical practice, however, treatment still relies mainly on empirical pharmacological measures intended to support the microcirculation, such as anticoagulation, antiplatelet therapy, and strategies to improve perfusion (Biermann et al., 2024). Systematic reviews have shown that commonly used antithrombotic regimens, including heparin, low-molecular-weight heparin, and aspirin, produce inconsistent effects on overall flap failure and pedicle thrombosis. These therapies may also increase the risk of complications, particularly bleeding and hematoma (Biermann et al., 2024; Lee and Mun, 2015; Liu et al., 2018; Dawoud et al., 2022). Although dextran-40 has shown some benefit in reducing partial flap necrosis in certain studies, its application is limited by safety concerns, especially pulmonary complications (Lin and Chen, 2024). At present, there is still no standardized pharmacological regimen specifically targeting mitochondrial injury, mtROS bursts, calcium overload, or disturbances in mitochondrial quality control. Taken together, flap ischemia-reperfusion treatment remains in transition, shifting from empirical microcirculatory support toward mechanism-based targeted intervention (Jia et al., 2025). However, clinical translation of mitochondria-targeted strategies remains limited by a key physiological barrier: effective drug delivery to the distal marginal zone of the flap, where severe hypoperfusion and no-reflow phenomena may restrict tissue penetration. This limitation may partly explain why mitochondria-targeted interventions remain largely preclinical, despite their strong mechanistic rationale. Future strategies should therefore combine mitochondrial targeting with local delivery platforms that improve tissue retention, penetration into poorly perfused regions, and sustained release within the ischemic flap microenvironment. Representative therapeutic strategies related to mitochondrial protection in flap ischemia-reperfusion injury are summarized in Table 1.

**TABLE 1:** Representative therapeutic strategies related to mitochondrial protection in flap ischemia-reperfusion injury.

Category	Representative drug/strategy	Main mitochondrial target	Main mechanism	Main effect on flap I/R injury	Model	Reference
Antioxidant	Metformin	Nrf2/HO-1	Alleviates oxidative stress	Improves skin flap survival	Rat random skin flap	Chen et al. (2024)
Antioxidant	TBHQ	Nrf2/HO-1	Enhances antioxidant enzyme activity	Improves blood supply	Rat random skin flap	Wang et al. (2024)
Antioxidant	Biliverdin	PI3K/Akt/Nrf2	Reduces ROS accumulation	Improves skin flap survival	Mouse random skin flap	Yao Z. et al. (2024)
Antioxidant	Ginsenoside Rg1	mtROS; JNK/ERK/p38	Alleviates mtROS and apoptosis	Improves skin flap survival	Rat random skin flap	Pan et al. (2025)
Cell-derived therapy	BMMSC-derived exosomes	mitochondrial stress response	Paracrine protection against I/R injury	Improves skin flap survival	Rat free abdominal flap I/R model	Niu et al. (2022)
Organelle therapy	UCMSC-derived mitochondrial transplantation	mitochondrial membrane stability	Supplements healthy mitochondria	Improves skin flap survival	Rat left inferior epigastric flap I/R model	Lee et al. (2024)
Antioxidant	Quercetin-loaded HMCeO_2_ hydrogel	mtROS	Continuous ROS scavenging with pro-repair support	Improves skin flap survival	Mouse random skin flap	Liu X. et al. (2025)
Biomaterial-enabled antioxidant	Pd@CeO_2_nanozyme	mtROS, multifaceted redox catalysis	Broad-spectrum ROS scavenging	Reduces inflammation/apoptosis/necrosis	Rat skin flap ischemia-reperfusion model	Zhou et al. (2025)
Bioenergetic restoration	Ginsenoside Rb1	Energy metabolis	Corrects metabolic disorder and alleviates mitochondrial dysfunction	Improves skin flap survival	Rat random skin flap	Huang et al. (2025)
Bioenergetic restoration	Empagliflozin	AMPK signaling	Activates energy-sensing pathway	Improves skin flap survival	Rat random skin flap	Yang et al. (2025e)
Bioenergetic regeneration	Sustained oxygen-releasing hydrogel	Mitochondrial biogenesis	Enhances mitochondrial biogenesis under mild hypoxia	Improves skin flap survival	Large rat random-pattern skin flap model	Jeon et al. (2025)
MQC restoration	Parkin-dependent mitophagy activation	Parkin, AMPK-TFEB, mitophagy	Promotes clearance of damaged mitochondria	Reduces oxidative/stress/apoptosis	Mouse random skin flap	Chen et al. (2022)
MQC modulation	ALDH2 activation	PINK1/Parkin-dependent mitophagy	Preserves mitochondrial homeostasis	Improves skin flap survival	Rat random skin flap	Zhou et al. (2023)
MQC/cell death crosstalk	Quercetin	SIRT1-regulated mitophagy, pyroptosis	Enhances mitophagy and suppresses pyroptosis	Improves skin flap survival	Rat random skin flap	Wang et al. (2026)
Programmed cell death	Thymoquinone	SIRT1/NF-κB/NLRP3	Reduces ROS accumulation and pyroptosis	Improves skin flap survival	Rat multi-territory perforator flap I/R model	Yang et al. (2025a)
Programmed cell death	Shuxuetong injection	TLR4/NF-κB/NLRP3	Inhibits pyroptosis-associated signaling	Improves skin flap survival	Rat random skin flap	Wang K. et al. (2025)
Programmed cell death	Exendin-4	GPX4, ferroptosis	Upregulates GPX4 and suppresses ferroptosis	Improves skin flap survival	Rat abdominal island skin flap ischemia-reperfusion model	Yu et al. (2024)
Programmed cell death	Osthole	Nrf2/SLC7A11/GPX4; NLRP3 /Caspase-1/GSDMD	Inhibits ferroptosis and alleviates pyroptosis	Improves skin flap survival	Rat random skin flap	Xu X. et al. (2025)

### 4.1 Protective strategies targeting oxidative stress

In flap ischemia-reperfusion injury, antioxidant intervention is a core protective approach. Its purpose is not simply to reduce total reactive oxygen species (ROS), but to limit sustained amplification of mitochondria-derived ROS and the resulting secondary injury to the electron transport chain and mitochondrial homeostasis (Jia et al., 2025). Existing studies show that conventional antioxidants, including metformin, tert-butylhydroquinone, and biliverdin, can reduce oxidative stress and improve flap perfusion and survival through activation of Nrf2-related antioxidant pathways (Chen et al., 2024; Wang et al., 2024; Yao Z. et al., 2024). However, most pharmacological and biomaterial-based antioxidant strategies in flap I/R have so far been supported by individual preclinical reports rather than independent replication across multiple laboratories. Therefore, these findings should be interpreted as promising but preliminary evidence. More recent work has increasingly turned to mitochondria-targeted antioxidant strategies. Ginsenoside Rg1, for example, has been shown to reduce mitochondrial oxidative stress and apoptosis, whereas mitochondrial transplantation from umbilical cord mesenchymal stem cells can suppress persistent mtROS generation and attenuate inflammatory injury by providing functional mitochondria (Lee et al., 2024; Pan et al., 2025). Nevertheless, mitochondrial transplantation remains difficult to translate clinically because maintaining organelle viability during extracellular isolation, storage, and delivery is technically challenging, and standardized protocols for mitochondrial source selection, quality control, dosing, timing, and delivery route are still lacking. Biomaterial-based approaches, including quercetin-loaded hollow mesoporous CeO_2_ nanoparticle hydrogels, Pd@CeO_2_ nanozymes, and sustained oxygen-releasing hydrogels, have also shown efficacy in promoting flap regeneration through continuous ROS scavenging, suppression of inflammation and apoptosis, and enhancement of mitochondrial biogenesis (Jeon et al., 2025; Liu X. et al., 2025; Zhou et al., 2025). However, these advanced strategies have so far been validated mainly in small-animal models, particularly rodent random-pattern flap models, and their relevance to human clinical free flaps should be interpreted cautiously because of substantial differences in flap size, vascular architecture, ischemic tolerance, reperfusion kinetics, and perioperative complexity. In addition, although CeO_2_- and Pd-based nanozymes exhibit strong catalytic antioxidant activity, their long-term tissue retention, degradation and clearance kinetics, potential chronic cytotoxicity, dose-dependent biodistribution, manufacturing consistency, and regulatory classification remain important barriers to clinical translation. Overall, antioxidant treatment in flap ischemia-reperfusion injury has progressed from conventional pharmacological scavenging toward integrated strategies that combine mtROS control, restoration of mitochondrial function, and support of tissue regeneration.

Nevertheless, the translational limitations of antioxidant therapy should be carefully considered. Experience from myocardial ischemia-reperfusion research shows that antioxidant or mitochondria-targeted strategies that are highly effective in preclinical models do not necessarily translate into clinical benefit. For example, the MITOCARE trial showed that TRO40303, a mitochondrial permeability transition pore-related cardioprotective compound, failed to reduce infarct size or improve myocardial salvage in patients with ST-segment elevation myocardial infarction undergoing primary percutaneous coronary intervention (Atar et al., 2015). This translational gap may be explained by several factors, including the very narrow therapeutic window of ROS bursts during early reperfusion, insufficient drug accumulation in mitochondria or ischemic tissue, differences between young and homogeneous animal models and clinically heterogeneous patients, interference from comorbidities and concomitant medications, and the dual role of ROS as both injurious mediators and physiological signaling molecules (Paillard et al., 2025; Chen H. et al., 2025; Ramachandra et al., 2020). Therefore, future antioxidant strategies for flap I/R should not simply aim at nonspecific ROS elimination, but should be evaluated with attention to mitochondrial targeting, timing of administration, local tissue delivery, dose-response relationships, and independently replicated efficacy in clinically relevant flap models.

### 4.2 Reducing calcium overload and inhibiting mPTP opening

In ischemia-reperfusion injury, mitochondrial Ca^2+^ overload and mitochondrial permeability transition pore (mPTP) opening are widely recognized as key events linking mitochondrial dysfunction to cell death (Murphy and Eisner, 2024; Morciano and Pinton, 2025). In flap I/R, these mechanisms are biologically plausible contributors to mitochondrial injury and distal tissue necrosis, although direct flap-specific evidence remains limited. During ischemia, ATP depletion and impaired ion pump activity drive sustained accumulation of cytosolic Ca^2+^ (Murphy and Eisner, 2024). After reperfusion, Ca^2+^ influx, the burst of mitochondrial reactive oxygen species (mtROS), and abnormal mitochondrial membrane permeability further aggravate mitochondrial Ca^2+^ overload (Murphy and Eisner, 2024). This sequence leads to mPTP opening, collapse of mitochondrial membrane potential, and release of pro-apoptotic factors (Morciano and Pinton, 2025). Current evidence largely supports mitochondrial Ca^2+^ dyshomeostasis and aberrant mPTP opening as major lethal mechanisms in reperfusion injury (Murphy and Eisner, 2024; Morciano and Pinton, 2025). In flap injury, secondary mitochondrial damage and calcium overload caused by oxidative bursts also contribute importantly to microcirculatory disturbance and expansion of distal necrosis (Jia et al., 2025; Wang Y. et al., 2025; Ye et al., 2023).

Current protective strategies are aimed mainly at upstream control of these events. Ginsenoside Rb1, for example, may indirectly reduce calcium overload-related injury by improving disordered energy metabolism and alleviating mitochondrial dysfunction (Huang et al., 2025). Mitochondrial transplantation from umbilical cord mesenchymal stem cells can also reduce oxidative stress, inflammation, and hypoxia-related injury by supplying functional mitochondria, thereby preserving membrane homeostasis and limiting secondary Ca^2+^ dyshomeostasis (Lee et al., 2024). In addition, suppression of the xanthine oxidase-related ROS burst may help reduce upstream triggers of mPTP opening (Jia et al., 2025). Taken together, the rationale for reducing calcium overload and inhibiting mPTP opening in flap I/R is already clear (Jia et al., 2025; Murphy and Eisner, 2024; Morciano and Pinton, 2025); but direct evidence remains limited. Further work is still needed, particularly on mitochondrial Ca^2+^ transport, cyclophilin D (CypD) regulation, and mPTP-targeted interventions (Jia et al., 2025; Morciano and Pinton, 2025).

### 4.3 Improving mitochondrial energy metabolism

In flap ischemia-reperfusion injury, metabolic protection against mitochondrial dysfunction centers on preserving oxidative phosphorylation, maintaining ATP supply, and improving mitochondrial respiratory function. Yeou et al. observed that ischemic flaps undergo ATP depletion, ionic imbalance, and metabolic acidosis during the early reperfusion phase (Yeou and Shin, 2026). These changes directly impair reparative capacity and distal flap survival. Studies have shown that GRK2 knockdown can increase mitochondrial ATP generation and ATP content, enhance respiratory chain complex activity, and improve oxygen consumption, thereby benefiting flap function and mitochondrial bioenergetic status (Wang et al., 2021). Likewise, ginsenoside Rb1 improves random flap survival through metabolic remodeling, correcting abnormalities in ATP, ADP, AMP, and tricarboxylic acid cycle-related metabolites while reducing lactate accumulation (Huang et al., 2025).

Beyond direct improvement of metabolic substrate utilization, promotion of mitochondrial biogenesis is also an important means of restoring bioenergetic function. Sustained oxygen-releasing hydrogels have been shown to enhance mitochondrial biogenesis, antioxidant capacity, and tissue regeneration. Empagliflozin can likewise improve flap survival through AMPK activation, suggesting that stimulation of energy-sensing and metabolic reprogramming pathways may help relieve mitochondrial bioenergetic dysfunction in flap ischemia-reperfusion injury (Jeon et al., 2025; Yang et al., 2025e). Overall, strategies to improve mitochondrial energy metabolism in flap ischemia-reperfusion injury are moving beyond simple correction of ischemia and hypoxia toward integrated approaches that enhance ATP production, support respiratory chain activity, and promote mitochondrial biogenesis.

### 4.4 Restoring mitochondrial quality control (MQC)

In flap ischemia-reperfusion injury associated with mitochondrial dysfunction, restoration of mitochondrial quality control depends on preserving the balance between recognition, clearance, and renewal of damaged mitochondria. This balance is necessary to avoid persistent accumulation of dysfunctional mitochondria, which can further amplify oxidative stress, inflammation, and cell death. Yan et al. noted that mitochondrial quality control includes mitophagy, mitochondrial biogenesis, and mitochondrial dynamics, and that coordinated regulation of these processes is required to maintain mitochondrial homeostasis and reparative capacity (Yan et al., 2025). Evidence from non-flap ischemia-reperfusion models further suggests that disruption of this network prevents timely renewal of damaged mitochondria and thereby worsens ischemia-reperfusion-related tissue injury (Zong et al., 2024).

In flap ischemia-reperfusion research, current evidence has focused mainly on regulation of mitophagy. Studies have shown that enhancement of Parkin-dependent mitophagy can reduce oxidative stress and apoptosis and improve random flap survival. By contrast, limiting excessive PINK1/Parkin-dependent mitophagy can also improve flap survival, indicating that its protective effect depends on appropriate intensity (Chen et al., 2022; Zhou et al., 2023; Wang et al., 2026). This apparent paradox may be explained by the timing and extent of mitophagy activation. During ischemia or early reperfusion, moderate mitophagy may be protective by selectively removing damaged, ROS-generating mitochondria and preserving mitochondrial quality. However, when PINK1/Parkin-dependent mitophagy is excessively or persistently activated during severe injury or late reperfusion, mitochondrial clearance may exceed mitochondrial biogenesis, leading to depletion of the functional mitochondrial pool, ATP insufficiency, and autophagy-associated cell death. Therefore, the therapeutic goal should not be simple activation or inhibition of mitophagy, but restoration of balanced mitophagic flux according to injury stage and mitochondrial renewal capacity. Promotion of mitochondrial biogenesis is another important route for restoring mitochondrial quality control. Sustained oxygen-releasing hydrogels have been shown to enhance mitochondrial biogenesis in random flap models while also improving antioxidant capacity, angiogenesis, and flap regeneration (Jeon et al., 2025). Mitochondrial transplantation has likewise emerged as a potential approach by directly replenishing functional mitochondria in injured tissue (Kubat et al., 2025). This strategy may help restore ATP production, redox balance, calcium buffering, and mitochondrial membrane potential, thereby contributing to reconstruction of mitochondrial homeostasis and complementing endogenous quality control mechanisms (Mukkala et al., 2025; Matiuto et al., 2025). One possible mechanism is that transplanted functional mitochondria are taken up by injured cells and incorporated into the endogenous mitochondrial network, thereby replenishing the respiratory pool, improving bioenergetic capacity, and reducing the burden on intrinsic mitophagy and biogenesis pathways.

Direct studies on fusion- and fission-related proteins in the flap field have remained relatively limited in recent years. Even so, existing evidence suggests that inhibition of aberrant Drp1 activation and excessive Drp1-dependent mitochondrial fission, rather than nonspecific suppression of physiological fission, can improve mitochondrial function and flap viability (Pan et al., 2025). Because excessive Drp1-mediated fission during reperfusion promotes mitochondrial fragmentation, apoptosis, and bioenergetic dysfunction, therapeutic strategies should focus on correcting pathological Drp1 overactivation while preserving basal fission required for mitochondrial quality control. This implies that restoration of mitochondrial dynamics is also an important part of mitochondria-targeted quality control strategies (Atici et al., 2023; Bai et al., 2023).

### 4.5 Inhibiting mitochondria-related programmed cell death

Apoptosis, pyroptosis, and ferroptosis are the forms of programmed cell death most closely linked to mitochondrial dysfunction in flap ischemia-reperfusion injury. Liu et al. pointed out that programmed cell death is not simply an accompanying event in flap I/R, but a key executional process connecting mitochondrial injury, inflammatory amplification, and expansion of tissue necrosis (Liu S. et al., 2025). In this setting, mitochondrial dysfunction can trigger apoptosis through membrane potential collapse, cytochrome c release, and caspase activation. It can also promote inflammasome activation through mtROS accumulation and mtDAMP release, thereby further driving pyroptosis and ferroptosis (Liu S. et al., 2025). Studies have shown that both ginsenoside Rg1 and Cu-DHM nanozymes improve flap survival by reducing mitochondrial oxidative stress and apoptosis (Pan et al., 2025; Zhao et al., 2025). Thymoquinone and Shuxuetong injection likewise lessen pyroptotic injury and improve flap necrosis by inhibiting NF-κB/NLRP3-related signaling (Yang et al., 2025a; Wang K. et al., 2025).

In recent years, ferroptosis and coordinated suppression of multiple death pathways have also received increasing attention. Exendin-4 inhibits ferroptosis through GPX4 upregulation, whereas osthole simultaneously suppresses ferroptosis via the Nrf2/SLC7A11/GPX4 axis and reduces pyroptosis through the NLRP3/caspase-1/GSDMD pathway. Quercetin can further improve flap survival by enhancing mitophagy and inhibiting pyroptosis through SIRT1 regulation (Yu et al., 2024; Xu P. et al., 2025; Wang et al., 2026). Overall, inhibition of mitochondria-related programmed cell death is important not only because it reduces individual death pathways, but also because it interrupts the self-amplifying cycle linking mitochondrial injury, inflammatory expansion, and cell death, thereby delaying tissue necrosis and supporting repair.

## 5 Conclusions and perspectives

Research on flap ischemia-reperfusion (I/R) injury has advanced substantially in recent years, yet effective and reliable clinical interventions remain limited. This gap suggests that the pathological basis of flap I/R is still not fully understood. Current evidence from flap studies, together with mechanistic insights from other ischemia-reperfusion models, indicates that mitochondria are among the earliest and most severely affected organelles in flap I/R injury and may act as a central node linking oxidative stress, calcium overload, metabolic dysfunction, inflammatory amplification, and programmed cell death. Accordingly, restoration of mitochondrial homeostasis has become an important direction for improving flap survival and tissue repair.

As discussed in this review, mitochondrial protection in flap I/R requires intervention at multiple levels. This includes not only controlling mitochondrial reactive oxygen species (mtROS), relieving calcium overload, and inhibiting mitochondrial permeability transition pore (mPTP) opening, but also improving energy metabolism, restoring mitochondrial quality control, and modulating programmed cell death. Disruption of mitochondrial homeostasis also influences flap outcome through its effects on several key effector cells. It can impair endothelial vascular reactivity, barrier integrity, and angiogenic capacity; sustain macrophages in a pro-inflammatory state; suppress fibroblast migration, proliferation, and extracellular matrix remodeling; and weaken maintenance of the contractile phenotype and vascular tone regulation in vascular smooth muscle cells.

A variety of pharmacological agents, natural products, functional biomaterials, stem cell-based therapies, organelle-based approaches, and mitochondrial transplantation strategies have shown protective effects against flap I/R injury at different levels. However, major barriers remain to clinical translation. These include defining the optimal intervention window at different stages of injury, clarifying how mitochondrial quality control pathways interact, and addressing the heterogeneity of cellular responses to mitochondrial damage. In addition, future studies should distinguish independently replicated findings from single-report observations, because many currently available mitochondria-targeted or antioxidant interventions in flap I/R remain at an early preclinical stage. Lessons from cardiac ischemia-reperfusion trials further suggest that promising antioxidant effects in animal models should be interpreted cautiously before clinical translation.

**FIGURE 3:**
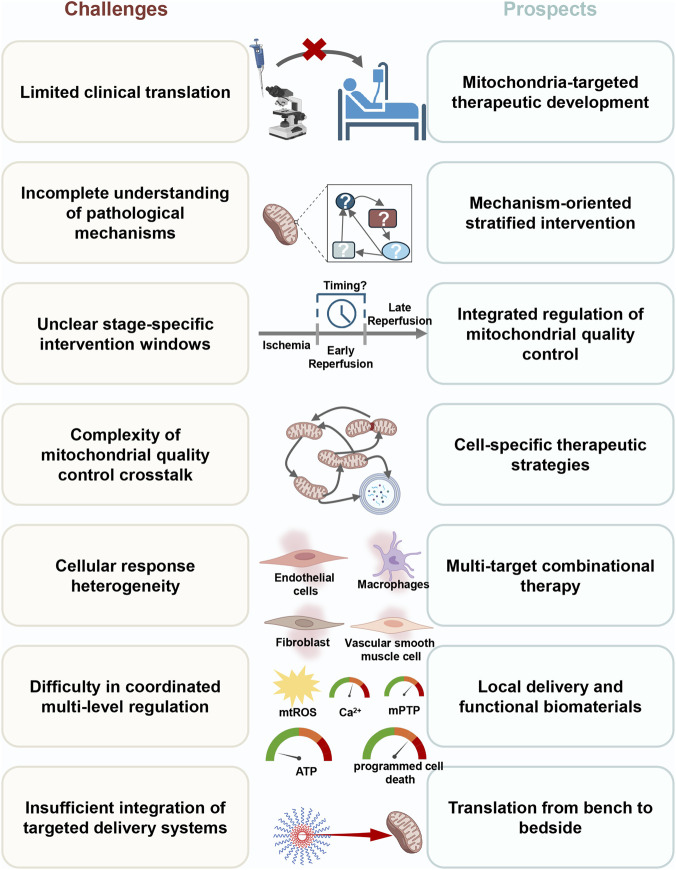
Challenges and prospects.

Further work is needed to clarify the intrinsic mechanisms that drive mitochondrial homeostasis imbalance in flap I/R. Such advances should support the coordinated development of mitochondria-targeted interventions and local delivery systems, and may ultimately provide new therapeutic strategies for improving flap survival and tissue repair (Figure 3).

## References

[B1] Alotaibi K. Arulkumaran N. Dyson A. Singer M. (2025). Therapeutic strategies to ameliorate mitochondrial oxidative stress in ischaemia-reperfusion injury: a narrative review. Clin. Sci. 139 (03), 259–280. 10.1042/cs20242074 PMC12204024 39899361

[B2] Ao-Di F. Han-Qing L. Xi-Zheng W. Ke Y. Hong-Xin G. Hai-xia Z. (2024). Advances in macrophage metabolic reprogramming in myocardial ischemia-reperfusion. Cell. Signal. 123, 111370. 10.1016/j.cellsig.2024.111370 39216681

[B3] Atar D. Arheden H. Berdeaux A. Bonnet J. L. Carlsson M. Clemmensen P. (2015). Effect of intravenous TRO40303 as an adjunct to primary percutaneous coronary intervention for acute ST-elevation myocardial infarction: MITOCARE study results. Eur. Heart Journal 36 (2), 112–119. 10.1093/eurheartj/ehu331 25179768

[B4] Atici A. E. Crother T. R. Noval Rivas M. (2023). Mitochondrial quality control in health and cardiovascular diseases. Front. Cell Developmental Biology 11, 1290046. 10.3389/fcell.2023.1290046 PMC10657886 38020895

[B5] Bai Y. Wu J. Yang Z. Wang X. Zhang D. Ma J. (2023). Mitochondrial quality control in cardiac ischemia/reperfusion injury: new insights into mechanisms and implications. Cell. Biology Toxicology 39 (1), 33–51. 10.1007/s10565-022-09716-2 35951200

[B6] Bansal R. Torres M. Hunt M. Wang N. Chatzopoulou M. Manchanda M. (2024). Role of the mitochondrial protein cyclophilin D in skin wound healing and collagen secretion. JCI Insight 9 (9), e169213. 10.1172/jci.insight.169213 38564292 PMC11141914

[B7] Bao Y. Hu C. Wang B. Liu X. Wu Q. Xu D. (2025). Mitochondrial reverse electron transport: mechanisms, pathophysiological roles, and therapeutic potential. Biol. (2079-7737) 14 (9), 1140. 10.3390/biology14091140 PMC12467400 41007285

[B8] Bertero E. Popoiu T.-A. Maack C. (2024). Mitochondrial calcium in cardiac ischemia/reperfusion injury and cardioprotection. Basic Research Cardiology 119 (4), 569–585. 10.1007/s00395-024-01060-2 38890208 PMC11319510

[B9] Biermann N. Chak J. C. Wiesmeier A. Klein S. M. Ruewe M. Spoerl S. (2024). Evidence-based approaches to anticoagulation in reconstructive microsurgery—a systematic literature review. Life 14 (1), 82. 10.3390/life14010082 38255697 PMC10817551

[B10] Bovill J. Huffman S. Cach G. Haffner Z. Deldar R. Abu El Hawa A. A. (2023). Propeller perforator flaps used for hand and digit reconstruction: a systematic review. J. Hand Microsurgery. 16 (2), 100035. 10.1055/s-0043-1768482 PMC11144646 38855530

[B11] Brooks N. A. Riar I. Klegeris A. (2026). Mitochondrial damage-associated molecular patterns: neuroimmunomodulators in central nervous system pathophysiology. Neural Regen. Res. 21 (4), 1322–1338. 10.4103/nrr.nrr-d-24-01459 40537002 PMC12407514

[B12] Bruqi K. Strappazzon F. (2025). NDP52 and its emerging role in pathogenesis. Cell. Death and Dis. 16 (1), 359. 10.1038/s41419-025-07668-z PMC12049512 40319017

[B13] Cai S. Zhao M. Zhou B. Yoshii A. Bugg D. Villet O. (2023). Mitochondrial dysfunction in macrophages promotes inflammation and suppresses repair after myocardial infarction. J. Clinical Investigation 133 (4), e159498. 10.1172/jci159498 PMC9927948 36480284

[B14] Cannito S. Giardino I. d’Apolito M. Pettoello-Mantovani M. Scaltrito F. Mangieri D. (2025). The multifaceted role of mitochondria in angiogenesis. Int. J. Mol. Sci. 26 (16), 7960. 10.3390/ijms26167960 40869281 PMC12386899

[B15] Cao L. Li Y. Smirnov A. Voshtani R. Wang T. Shao C. (2025). PGC-1α: key regulator of mitochondrial biogenesis and cellular differentiation in metabolic and regenerative tissues. Cell. and Biosci. 16, 9. 10.1186/s13578-025-01519-2 PMC12853593 41456074

[B16] Chai Z. Zhou Y. Hossain S. Huynh K. Hajimirzaei S. Zhang Y. (2025). Cardiolipin’s multifaceted role in immune response: a focus on interacting proteins. Front. Immunol. 16, 1680326. 10.3389/fimmu.2025.1680326 41438747 PMC12719303

[B17] Chen Z. Wu H. Yang J. Li B. Ding J. Cheng S. (2022). Activating Parkin-dependent mitophagy alleviates oxidative stress, apoptosis, and promotes random-pattern skin flaps survival. Commun. Biology 5 (1), 616. 10.1038/s42003-022-03556-w PMC9217959 35732814

[B18] Chen Y. Cheng R. Lu W. Fan Y. Yu Y. Huang L. (2024). Metformin promotes the survival of random skin flaps *via* the activation of Nrf2/HO-1 signaling. Chemico-Biological Interact. 401, 111188. 10.1016/j.cbi.2024.111188 39121897

[B19] Chen K.-Q. Tang W.-R. Liu X. (2025). Research and progress of cGAS/STING/NLRP3 signaling pathway: a mini review. Front. Immunol. 16, 1594133. 10.3389/fimmu.2025.1594133 40463371 PMC12129981

[B20] Chen C. Dai G. Fan M. Wang X. Niu K. Gao W. (2025). Mitochondria-associated endoplasmic reticulum membranes and myocardial ischemia: from molecular mechanisms to therapeutic strategies. J. Transl. Med. 23 (1), 277. 10.1186/s12967-025-06262-3 40050915 PMC11884070

[B21] Chen H. Tang Y. Ren P. Wu W. (2025). The unmet promise: a critical review of antioxidant strategies in myocardial ischemia-reperfusion injury and the path towards precision medicine. Front. Pharmacol. 16, 1693441. 10.3389/fphar.2025.1693441 41293252 PMC12640823

[B22] Cipriano A. Viviano M. Feoli A. Milite C. Sarno G. Castellano S. (2023). NADPH oxidases: from molecular mechanisms to current inhibitors. J. Medicinal Chemistry 66 (17), 11632–11655. 10.1021/acs.jmedchem.3c00770 PMC10510401 37650225

[B23] Civiletto G. Brunetti D. Lizzo G. Muller K. Jacot G. E. Daskalaki I. (2025). Herbal terpenoids activate autophagy and mitophagy through modulation of bioenergetics and protect from metabolic stress, sarcopenia and epigenetic aging. Nat. Aging 5 (10), 2003–2021. 10.1038/s43587-025-00957-4 40993327 PMC12532568

[B24] Dawoud B. Kent S. Tabbenor O. Markose G. Java K. Kyzas P. (2022). Does anticoagulation improve outcomes of microvascular free flap reconstruction following head and neck surgery: a systematic review and meta-analysis. Br. J. Oral Maxillofac. Surg. 60 (10), 1292–1302. 10.1016/j.bjoms.2022.07.016 36328862

[B25] Drysch M. Fiedler A. Kurbacher T. Schmidt S. V. Reinkemeier F. Puscz F. (2025). Ischemia–reperfusion injury in free flaps: molecular mechanisms and protective effects of remote ischemic preconditioning. J. Cell. Mol. Med. 29 (15), e70739. 10.1111/jcmm.70739 40771071 PMC12329344

[B26] D’Egidio F. Qosja E. Ammannito F. Topi S. d’Angelo M. Cimini A. (2025). Antioxidant and anti-inflammatory defenses in Huntington’s disease: roles of NRF2 and PGC-1α, and therapeutic strategies. Life 15 (4), 577. 10.3390/life15040577 40283130 PMC12028459

[B27] Fang W.-C. Lan C.-C. E. (2023). The epidermal keratinocyte as a therapeutic target for management of diabetic wounds. Int. J. Mol. Sci. 24 (5), 4290. 10.3390/ijms24054290 36901720 PMC10002069

[B28] Fang S. Huang W. Qu X. Chai W. (2025). The mitochondria as a potential therapeutic target in cerebral I/R injury. Front. Neurosci. 18, 1500647. 10.3389/fnins.2024.1500647 39844858 PMC11752919

[B29] Grossini E. Venkatesan S. Ola Pour M. M. (2025). Mitochondrial dysfunction in endothelial cells: a key driver of organ disorders and aging. Antioxidants 14 (4), 372. 10.3390/antiox14040372 40298614 PMC12024085

[B30] Guo Z. Tian Y. Gao J. Zhou B. Zhou X. Chang X. (2024). Enhancement of mitochondrial homeostasis: a novel approach to attenuate hypoxic myocardial injury. Int. Journal Medical Sciences 21 (15), 2897–2911. 10.7150/ijms.103986 PMC11610329 39628681

[B31] Hao Y. Chen F. Ren X. Huang X. Zhou X. (2025). Harnessing mitochondrial biogenesis to combat acute kidney injury: current insights and future directions. Genes. and Dis. 12, 101645. 10.1016/j.gendis.2025.101645 PMC12357249 40821125

[B32] He Y. Ren S. Liu C. Zheng X. Zhu C. (2025). Targeting mitochondrial quality control for myocardial ischemia-reperfusion injury. Mitochondrion 84, 102046. 10.1016/j.mito.2025.102046 40419068

[B33] Huang Y. Liu Y. Meng F. Wijaya W. A. Cao C. (2025). 1H NMR metabonomic analysis of serum and flap tissue on the effect of ginsenoside Rb1 on survival of random pattern skin flaps in rats. Sci. Rep. 15 (1), 7416. 10.1038/s41598-025-91798-z 40033034 PMC11876603

[B34] Hwang J.-H. Kim K. M. Oh H. T. Yoo G. D. Jeong M. G. Lee H. (2022). TAZ links exercise to mitochondrial biogenesis *via* mitochondrial transcription factor A. Nat. Communications 13 (1), 653. 10.1038/s41467-022-28247-2 PMC8814203 35115527

[B35] Jeon S. Jeong S.-H. Lee M. H. Seo J. W. Kim D. S. Bassous N. J. (2025). Sustained oxygen-releasing hydrogel implants enhance flap regeneration by promoting mitochondrial biogenesis under mild hypoxia. Bioact. Mater. 51, 559–574. 10.1016/j.bioactmat.2025.04.010 40503164 PMC12155817

[B36] Jia W. Wei X. Gong X. (2025). Mechanism of xanthine oxidase in flap ischemia-reperfusion injury and advances in targeted therapy: a mini review. Front. Physiology 16, 1705704. 10.3389/fphys.2025.1705704 PMC12682682 41367389

[B37] Jiang R.-Q. Li Q.-Q. Sheng R. (2023). Mitochondria associated ER membranes and cerebral ischemia: molecular mechanisms and therapeutic strategies. Pharmacol. Research 191, 106761. 10.1016/j.phrs.2023.106761 37028777

[B38] Kirshenbaum L. A. Dhingra R. Bravo-Sagua R. Lavandero S. (2024). DIAPH1-MFN2 interaction decreases the endoplasmic reticulum-mitochondrial distance and promotes cardiac injury following myocardial ischemia. Nature Communications 15 (1), 1469. 10.1038/s41467-024-45560-0 PMC10874398 38368414

[B39] Klein D. (2025). The vascular endothelium as decision maker in lung injury. Front. Cell. Dev. Biol. 13, 1564627. 10.3389/fcell.2025.1564627 40692751 PMC12277298

[B40] Kopych V. Da Costa A. D. S. Park K. (2025). Endothelial dysfunction in atherosclerosis: experimental models and therapeutics. Biomaterials Res. 29, 0252. 10.34133/bmr.0252 PMC12504828 41069871

[B41] Kubat G. B. Picone P. Tuncay E. Aryan L. Girgenti A. Palumbo L. (2025). Biotechnological approaches and therapeutic potential of mitochondria transfer and transplantation. Nat. Commun. 16 (1), 5709. 10.1038/s41467-025-61239-6 40593725 PMC12217326

[B42] Kumar M. Sharma S. Kumar J. Barik S. Mazumder S. (2024). Mitochondrial electron transport chain in macrophage reprogramming: potential role in antibacterial immune response. Curr. Research Immunology 5, 100077. 10.1016/j.crimmu.2024.100077 PMC10987323 38572399

[B43] Lee K.-T. Mun G.-H. (2015). The efficacy of postoperative antithrombotics in free flap surgery: a systematic review and meta-analysis. Plastic Reconstructive Surgery 135 (4), 1124–1139. 10.1097/PRS.0000000000001100 25811576

[B44] Lee C. Y. Khan G. Hyun D. Y. Kim S. H. Park E. S. (2024). Effect of umbilical cord mesenchymal stem cell‐derived mitochondrial transplantation on ischemia‐reperfusion injury in a rat model. Skin Res. Technol. 30 (9), e70022. 10.1111/srt.70022 39221632 PMC11367251

[B45] Li Y. Wang H.-b. Cao J.-l. Zhang W. j. Wang H. l. Xu C. h. (2024). Proteomic analysis of mitochondria associated membranes in renal ischemic reperfusion injury. J. Transl. Med. 22 (1), 261. 10.1186/s12967-024-05021-0 38461333 PMC10925013

[B46] Li Y. Liu Y. Wu H. Zhang S. Liu D. Huang X. (2025). Lysosomal homeostasis regulates myocardial ischemia-reperfusion injury through autophagy pathway. Sci. Rep. 15 (1), 36952. 10.1038/s41598-025-20853-6 41125732 PMC12546593

[B47] Lin Y.-E. Chen M.-C. (2024). Dextran-40 reduces partial flap failure: a systematic review and meta-analysis for antithrombotics after free flaps. Plastic Reconstr. Surgery–Global Open 12 (5), e5812. 10.1097/gox.0000000000005812 PMC11095965 38752217

[B48] Liu J. Shi Q. Yang S. Liu B. Guo B. Xu J. (2018). Does postoperative anticoagulation therapy lead to a higher success rate for microvascular free-tissue transfer in the head and neck? A systematic review and meta-analysis. J. Reconstr. Microsurg. 34 (02), 087–094. 10.1055/s-0037-1606346 29036750

[B49] Liu L. Li Y. Chen G. Chen Q. (2023). Crosstalk between mitochondrial biogenesis and mitophagy to maintain mitochondrial homeostasis. J. Biomedical Science 30 (1), 86. 10.1186/s12929-023-00975-7 PMC10568841 37821940

[B50] Liu M. Li S. Yin M. Li Y. Chen J. Chen Y. (2024). Pinacidil ameliorates cardiac microvascular ischemia–reperfusion injury by inhibiting chaperone-mediated autophagy of calreticulin. Basic Res. Cardiol. 119 (1), 113–131. 10.1007/s00395-023-01028-8 38168863 PMC10837255

[B51] Liu Z. Chen D.-H. Lin Z.-H. Wang Z. Y. Peng H. Liu R. T. (2025). *In-situ* sprayed platelet-derived small extracellular vesicles for the skin flap survival by reducing PANoptosis. Biomaterials 316, 123001. 10.1016/j.biomaterials.2024.123001 39671720

[B52] Liu S. Xiong X. Chen L. Hu J. Luo P. Ou Z. (2025). Targeting programmed cell death in flap ischemia/reperfusion injury. Biomolecules 15 (7), 911. 10.3390/biom15070911 40723782 PMC12292106

[B53] Liu G. Dai Y. Fu C. Lv X. Qin J. Xie J. (2025). Macrophage polarization in myocardial ischemia‒reperfusion injury: pathophysiology and therapeutic targets. Drug Des. Dev. Ther. 19, 6519–6541. 10.2147/dddt.s516001 PMC12323797 40766819

[B54] Liu Y. Jin W. Zhou H. Wang X. Ren H. Yang X. (2025). Role of mitochondrial Ca^2+^ in stroke: from molecular mechanism to treatment strategy. Mol. Med. Rep. 271. 10.3892/mmr.2025.13636 40747662 PMC12332370

[B55] Liu M. Yin Z. Li B. Qiu J. Zhang D. Wang R. (2025). Targeting Drp1 in cerebral ischemia–reperfusion injury: mechanisms and therapeutic implications. CNS Neurosci. and Ther. 31 (8), e70590. 10.1111/cns.70590 40878129 PMC12394188

[B56] Liu X. Ju Y. Yang P. Shen N. Shao Y. Yang A. (2025). Enhanced hydrogel loading of quercetin-loaded hollow mesoporous cerium dioxide nanoparticles for skin flap survival. Mater. Today Bio 30, 101432. 10.1016/j.mtbio.2024.101432 PMC11745961 39839491

[B57] Lu X. Wang Y. Geng N. Zou Z. Feng X. Wang Y. (2024). Dysregulated mitochondrial calcium causes spiral artery remodeling failure in preeclampsia. Hypertension 81 (11), 2368–2382. 10.1161/hypertensionaha.124.23046 39291377

[B58] Luo Z. Yao J. Wang Z. Xu J. (2023). Mitochondria in endothelial cells angiogenesis and function: current understanding and future perspectives. J. Translational Medicine 21 (1), 441. 10.1186/s12967-023-04286-1 PMC10324193 37407961

[B59] Luo X. Zhang S. Wang L. Li J. (2024). Pathological roles of mitochondrial dysfunction in endothelial cells during the cerebral no-reflow phenomenon: a review. Medicine 103 (51), e40951. 10.1097/md.0000000000040951 39705421 PMC11666140

[B60] Matarazzo S. Corsini B. Cozzi S. Tellarini A. Valdatta L. Paganini F. (2025). Propeller flaps for acute lower limb reconstruction after trauma: evidence from a systematic review. J. Clin. Med. 14 (17), 6288. 10.3390/jcm14176288 40944046 PMC12428917

[B61] Matiuto N. Applewhite B. Habash N. Martins A. Wang B. Jiang B. (2025). Harnessing mitochondrial transplantation to target vascular inflammation in cardiovascular health. JACC Basic Transl. Sci. 10 (8), 101331. 10.1016/j.jacbts.2025.101331 40714674 PMC12314333

[B62] Morciano G. Pinton P. (2025). Modulation of mitochondrial permeability transition pores in reperfusion injury: mechanisms and therapeutic approaches. Eur. J. Clin. Investigation 55 (1), e14331. 10.1111/eci.14331 PMC11628652 39387139

[B63] Mukkala A. N. David B. A. Ailenberg M. Liang J. Vaswani C. M. Karakas D. (2025). Mitochondrial transplantation: a novel therapy for liver ischemia/reperfusion injury. Ann. Surg. 281 (6), 1032–1047. 10.1097/sla.0000000000006655 39912224

[B64] Murphy E. Eisner D. A. (2024). How does mitochondrial Ca^2+^ change during ischemia and reperfusion? Implications for activation of the permeability transition pore. J. General Physiology 157 (1), e202313520. 10.1085/jgp.202313520 PMC11657230 39699565

[B65] Niu Q. Yang Y. Li D. Guo W. Wang C. Xu H. (2022). Exosomes derived from bone marrow mesenchymal stem cells alleviate ischemia-reperfusion injury and promote survival of skin flaps in rats. Life 12 (10), 1567. 10.3390/life12101567 36295004 PMC9604753

[B66] Pagliaro P. Penna C. Femminò S. Welt F. G. P. (2026). Insights in ischemia/reperfusion injury and cardioprotection: neglected and emerging pathways and therapeutic targets for a personalized therapy. Basic Res. Cardiol. 121, 1–30. 10.1007/s00395-026-01167-8 41826738 PMC13186818

[B67] Paik S. Kim J. K. Shin H. J. Park E. J. Kim I. S. Jo E. K. (2025). Updated insights into the molecular networks for NLRP3 inflammasome activation. Cell. and Molecular Immunology 22 (6), 563–596. 10.1038/s41423-025-01284-9 PMC12125403 40307577

[B68] Paillard M. Abdellatif M. Andreadou I. Bär C. Bertrand L. Brundel B. J. (2025). Mitochondrial targets in ischaemic heart disease and heart failure, and their potential for a more efficient clinical translation. A scientific statement of the ESC working group on cellular biology of the heart and the ESC working group on myocardial function. Eur. Journal Heart Failure 27 (9), 1720–1736. 10.1002/ejhf.3674 PMC12502468 40320260

[B69] Pan H. Xu P. Wang K. Yang J. Song W. Wang A. (2025). Ginsenoside Rg1 promotes skin flap survival by alleviating mitochondrial oxidative stress and apoptosis *via* the JNK/ERK/p38 pathway. J. Ginseng Res. 50, 100934. 10.1016/j.jgr.2025.100934 41788575 PMC12959292

[B70] Pearce W. J. (2024). Mitochondrial influences on smooth muscle phenotype. Am. J. Physiol. Cell Physiol. 326 (2), C442–C448. 10.1152/ajpcell.00354.2023 38009196 PMC11932527

[B71] Pham L. Arroum T. Wan J. Pavelich L. Bell J. Morse P. T. (2024). Regulation of mitochondrial oxidative phosphorylation through tight control of cytochrome c oxidase in health and disease–implications for ischemia/reperfusion injury, inflammatory diseases, diabetes, and cancer. Redox Biology 78, 103426. 10.1016/j.redox.2024.103426 39566165 PMC11617887

[B72] Qin H.-L. Bao J.-H. Tang J.-J. Xu D. Y. Shen L. (2023). Arterial remodeling: the role of mitochondrial metabolism in vascular smooth muscle cells. Am. J. Physiology-Cell Physiology 324 (1), C183–C192. 10.1152/ajpcell.00074.2022 36468843

[B73] Ramachandra C. J. Hernandez-Resendiz S. Crespo-Avilan G. E. Lin Y. H. Hausenloy D. J. (2020). Mitochondria in acute myocardial infarction and cardioprotection. EBioMedicine 57, 102884. 10.1016/j.ebiom.2020.102884 32653860 PMC7355051

[B74] Rius-Pérez S. Pérez S. Toledano M. B. Sastre J. (2023). Mitochondrial reactive oxygen species and lytic programmed cell death in acute inflammation. Antioxidants and Redox Signaling 39 (10-12), 708–727. 10.1089/ars.2022.0209 37450339 PMC10619893

[B75] Rozich E. Ozkurede U. Pakkiriswami S. Gemilere R. Azarin S. M. Liu J. C. (2025). Mitochondrial oxidative stress, calcium and dynamics in cardiac ischaemia‐reperfusion injury. J. Physiol. 10.1113/JP287770 PMC13371218 40448972

[B76] Serra P. L. Boriani F. Khan U. Atzeni M. Figus A. (2024). Rate of free flap failure and return to the operating room in lower limb reconstruction: a systematic review. J. Clin. Med. 13 (15), 4295. 10.3390/jcm13154295 39124562 PMC11313376

[B77] Shan W. Liu Y. Tang R. Li H. Yang H. Lin L. (2025). Targeting mitochondrial autophagy for anti-aging. Cell. Death Discov. 12, 78. 10.1038/s41420-025-02913-y 41444209 PMC12876910

[B78] Shen L. Gan Q. Yang Y. Reis C. Zhang Z. Xu S. (2021). Mitophagy in cerebral ischemia and ischemia/reperfusion injury. Front. Aging Neuroscience 13, 687246. 10.3389/fnagi.2021.687246 PMC8217453 34168551

[B79] Singh D. (2025). Mitochondrial permeability transition pore (mPTP) as a target for drug delivery systems: challenges and opportunities. Health Nanotechnol. 1 (1), 19. 10.1186/s44301-025-00019-z

[B80] Suzuki Y. (2025). Ca^2+^ microdomains in vascular smooth muscle cells: roles in vascular tone regulation and hypertension. J. Pharmacol. Sci. 158 (1), 59–67. 10.1016/j.jphs.2025.03.008 40121058

[B81] Uoselis L. Nguyen T. N. Lazarou M. (2023). Mitochondrial degradation: mitophagy and beyond. Mol. Cell 83 (19), 3404–3420. 10.1016/j.molcel.2023.08.021 37708893

[B82] Wang X. He B. (2024). Endothelial dysfunction: molecular mechanisms and clinical implications. MedComm 5 (8), e651. 10.1002/mco2.651 39040847 PMC11261813

[B83] Wang Y. Wu Y. Zhou M. Wang P. Luo J. Rui Y. (2021). GRK2 deletion improves the function of skin flap following ischemia-reperfusion injury by regulating Drp1. Am. J. Transl. Res. 13 (1), 223–233. Available online at: https://www.ajtr.org/article/AJTR0116281. 33527020 PMC7847531

[B84] Wang S. Long H. Hou L. Feng B. Ma Z. Wu Y. (2023). The mitophagy pathway and its implications in human diseases. Signal Transduction Targeted Therapy 8 (1), 304. 10.1038/s41392-023-01503-7 37582956 PMC10427715

[B85] Wang K. Wang A. Deng J. Yang J. Chen G. Chen Q. (2024). Tert‐butylhydroquinone promotes skin flap survival by inhibiting oxidative stress mediated by the Nrf2/HO‐1 signalling pathway. Br. J. Pharmacol. 181 (23), 4845–4858. 10.1111/bph.17321 39233316

[B86] Wang K. Xu P. Wang A. Deng J. Yang J. Song W. (2025). Shuxuetong injection promotes skin flap survival by inhibiting pyroptosis mediated by the TLR4/NF-κB/NLRP3 signaling pathway. J. Ethnopharmacol. 355, 120749. 10.1016/j.jep.2025.120749 41106569

[B87] Wang Y. Lou J. Dai R. Chen L. Wang Y. Wang Y. (2025). Far-infrared irradiation suppresses pyroptosis in ischemic flaps through TRPV3-mediated activation of the A2AR/EPAC1/Rap1 signaling pathway. Cell. Signal. Physiol. Cell Physiol 10.1016/j.cellsig.2025.112299 41354356

[B88] Wang A. Chen S. Yang J. Deng J. Song W. Xu P. (2026). Quercetin enhances skin flap survival *via* SIRT1-regulated mitophagy promotion and pyroptosis inhibition. J. Ethnopharmacol. 364, 121525. 10.1016/j.jep.2026.121525 41831738

[B89] Weidman A. A. Parikh R. P. (2026). Discussion: mastectomy skin flap necrosis after implant-based breast reconstruction: intraoperative predictors and indocyanine green angiography. Plastic Reconstr. Surg. 157 (1), 29–30. 10.1097/prs.0000000000012402 41427756

[B90] Xiong Y. Knoedler S. Alfertshofer M. Kim B. S. Jiang D. Liu G. (2025). Mechanisms and therapeutic opportunities in metabolic aberrations of diabetic wounds: a narrative review. Cell. Death and Dis. 16 (1), 341. 10.1038/s41419-025-07583-3 PMC12032273 40280905

[B91] Xu S. Wang Z. Guo F. Zhang Y. Peng H. Zhang H. (2024). Mitophagy in ischemic heart disease: molecular mechanisms and clinical management. Cell. Death and Dis. 15 (12), 934. 10.1038/s41419-024-07303-3 PMC11685431 39737905

[B92] Xu X. Pang Y. Fan X. (2025). Mitochondria in oxidative stress, inflammation and aging: from mechanisms to therapeutic advances. Signal Transduct. Target. Ther. 10 (1), 190. 10.1038/s41392-025-02253-4 40500258 PMC12159213

[B93] Xu P. Wang A. Xiao Z. Song W. Pan H. Yang J. (2025). Osthole promotes skin flap survival by inhibiting ferroptosis and alleviating pyroptosis. J. Ethnopharmacol. 358, 121058. 10.1016/j.jep.2025.121058 41406561

[B94] Xue K. Zhang G. Zhou Y. Wang K. Yao Z. Chen J. (2023). Nuciferine improves random skin flap survival *via* TFEB-Mediated activation of autophagy-lysosomal pathway. Int. Immunopharmacol. 119, 110204. 10.1016/j.intimp.2023.110204 37126988

[B95] Yan R. Zhang Y. Li Z. Li K. Man J. Yang L. (2025). Current perspectives and trends on the role of mitochondria in renal ischemia-reperfusion injury from 2005 to 2024: a bibliometric analysis and literature review. Front. Physiology 16, 1705821. 10.3389/fphys.2025.1705821 PMC12727442 41451126

[B96] Yang Z. Yoshii S. R. Sakai Y. Zhang J. Chino H. Knorr R. L. (2024). Autophagy adaptors mediate Parkin-dependent mitophagy by forming sheet-like liquid condensates. EMBO J. 43 (22), 5613–5634. 10.1038/s44318-024-00272-5 39420095 PMC11574277

[B97] Yang J. Zhang H. Ni L. He J. (2025a). Thymoquinone alleviates the accumulation of ROS and pyroptosis and promotes perforator skin flap survival through SIRT1/NF-κB pathway. Front. Pharmacol. 16, 1567762. 10.3389/fphar.2025.1567762 40201684 PMC11975933

[B98] Yang M. Liu B. Chen B. Shen Y. (2025b). Cerebral ischemia-reperfusion injury: mechanisms and promising therapies. Front. Pharmacology 16, 1613464. 10.3389/fphar.2025.1613464 PMC12323178 40766753

[B99] Yang L. Zhang Y. Chai Z. Zhou Y. Li Z. Wei Y. (2025). Regulation of pyroptosis by NF-κB signaling. Front. Cell. Death 3, 1503799. 10.3389/fceld.2024.1503799

[B100] Yang M. Sun L. Feng X. Xu W. (2025d). Mitochondrial transcription factor a as a guardian of mitochondrial integrity and emerging therapeutic target in human diseases: a review. Int. J. Biol. Macromol. 319, 145706. 10.1016/j.ijbiomac.2025.145706 40609926

[B101] Yang J. Ye W. Wang K. Wang A. Deng J. Chen G. (2025e). Empagliflozin promotes skin flap survival by activating AMPK signaling pathway. Eur. Journal Pharmacology 987, 177207. 10.1016/j.ejphar.2024.177207 39694175

[B102] Yao B.-F. Luo X.-J. Peng J. (2024). A review for the correlation between optic atrophy 1-dependent mitochondrial fusion and cardiovascular disorders. Int. J. Biol. Macromol. 254, 127910. 10.1016/j.ijbiomac.2023.127910 37939779

[B103] Yao Z. Xue K. Chen J. Zhang Y. Zhang G. Zheng Z. (2024). Biliverdin improved angiogenesis and suppressed apoptosis *via* PI3K/Akt-mediated Nrf2 antioxidant system to promote ischemic flap survival. Free Radic. Biol. Med. 225, 35–52. 10.1016/j.freeradbiomed.2024.09.042 39332540

[B104] Yap C. Wanga S. Wuest R. C. van Os B. W. Pijls M. M. Keijzer S. (2024). Doxycycline induces mitochondrial dysfunction in aortic smooth muscle cells. Vasc. Pharmacol. 154, 107279. 10.1016/j.vph.2024.107279 38272196

[B105] Ye H. Li F. Shen Y. Wu X. Zhao L. Zhang H. (2023). Rosuvastatin promotes survival of random skin flaps through AMPK-mTOR pathway-induced autophagy. Int. Immunopharmacol. 118, 110059. 10.1016/j.intimp.2023.110059 37001384

[B106] Yeou S. H. Shin Y. S. (2026). Regenerative approach for improving flap survival: perspective of angiogenesis. Biomimetics 11 (3), 186. 10.3390/biomimetics11030186 41892109 PMC13023543

[B107] Yu W. Lu J. Huang X. Zhuang H. An Y. Zhang M. (2024). Exendin-4 promotes ischemia-reperfusion flap survival by upregulating Gpx4 to inhibit ferroptosis. Eur. J. Pharmacol. 984, 177029. 10.1016/j.ejphar.2024.177029 39366501

[B108] Zaccaron R. P. Mendes C. da Costa C. Silveira P. C. L. Rezin G. T. (2024). Skin metabolism in obesity: a narrative review. Wound Repair Regen. 32 (6), 1022–1027. 10.1111/wrr.13223 39318160

[B109] Zhang C. Chang X. Zhao D. He Y. Dong G. Gao L. (2025). Mitochondria and myocardial ischemia/reperfusion injury: effects of Chinese herbal medicine and the underlying mechanisms. J. Pharm. Analysis 15 (2), 101051. 10.1016/j.jpha.2024.101051 PMC11808734 39931135

[B110] Zhang T. Li Z. Xu Y. Xu C. Wang H. Rui T. (2025). Regulation of mitochondrial dynamics in cardiomyocytes: implications for cardiac health and disease. Front. Cell. Dev. Biol. 13, 1652683. 10.3389/fcell.2025.1652683 41000071 PMC12457398

[B111] Zhao X. Zhang S. Wang M. Li Q. Wei X. Chen X. L. (2025). Cu-DHM nanozymes treat flap ischemia-reperfusion injury by amplifying immune modulation in a cascade manner and inhibiting cell apoptosis. Bioact. Mater. 51, 720–739. 10.1016/j.bioactmat.2025.06.036 40641838 PMC12242327

[B112] Zhou T. Wang X. Wang K. Lin Y. Meng Z. Lan Q. (2023). Activation of aldehyde dehydrogenase-2 improves ischemic random skin flap survival in rats. Front. Immunology 14, 1127610. 10.3389/fimmu.2023.1127610 PMC10335790 37441072

[B113] Zhou L. Sun J. Lu T. Zhang X. Wang M. Xu Y. (2025). Electron-injected pd@ CeO_2_ nanozymes for multifaceted ROS scavenging and protection against ischemia-reperfusion injury in skin flaps. J. Nanobiotechnology 23 (1), 1–15. 10.1186/s12951-025-03775-3 41116175 PMC12538877

[B114] Zong Y. Li H. Liao P. Chen L. Pan Y. Zheng Y. (2024). Mitochondrial dysfunction: mechanisms and advances in therapy. Signal Transduction Targeted Therapy 9 (1), 124. 10.1038/s41392-024-01839-8 38744846 PMC11094169

